# A uni-extension study on the ultimate material strength and extreme extensibility of atherosclerotic tissue in human carotid plaques

**DOI:** 10.1016/j.jbiomech.2015.09.037

**Published:** 2015-11-05

**Authors:** Zhongzhao Teng, Jiaxuan Feng, Yongxue Zhang, Michael P.F. Sutcliffe, Yuan Huang, Adam J. Brown, Zaiping Jing, Qingsheng Lu, Jonathan H. Gillard

**Affiliations:** a Department of Radiology, University of Cambridge, UK; b Department of Engineering, University of Cambridge, UK; c Department of Vascular Surgery, Changhai Hospital, Shanghai, China; d Division of Cardiovascular Medicine, University of Cambridge, UK

**Keywords:** Atherosclerosis, Ultimate strength, Extensibility, Fibrous cap, Lipid core, Hemorrhage

## Abstract

Atherosclerotic plaque rupture occurs when mechanical loading exceeds its material strength. Mechanical analysis has been shown to be complementary to the morphology and composition for assessing vulnerability. However, strength and stretch thresholds for mechanics-based assessment are currently lacking. This study aims to quantify the ultimate material strength and extreme extensibility of atherosclerotic components from human carotid plaques. Tissue strips of fibrous cap, media, lipid core and intraplaque hemorrhage/thrombus were obtained from 21 carotid endarterectomy samples of symptomatic patients. Uni-extension test with tissue strips was performed until they broke or slid. The Cauchy stress and stretch ratio at the peak loading of strips broken about 2 mm away from the clamp were used to characterize their ultimate strength and extensibility. Results obtained indicated that ultimate strength of fibrous cap and media were 158.3 [72.1, 259.3] kPa (Median [Inter quartile range]) and 247.6 [169.0, 419.9] kPa, respectively; those of lipid and intraplaque hemorrhage/thrombus were 68.8 [48.5, 86.6] kPa and 83.0 [52.1, 124.9] kPa, respectively. The extensibility of each tissue type were: fibrous cap – 1.18 [1.10, 1.27]; media – 1.21 [1.17, 1.32]; lipid – 1.25 [1.11, 1.30] and intraplaque hemorrhage/thrombus – 1.20 [1.17, 1.44]. Overall, the strength of fibrous cap and media were comparable and so were lipid and intraplaque hemorrhage/thrombus. Both fibrous cap and media were significantly stronger than either lipid or intraplaque hemorrhage/thrombus. All atherosclerotic components had similar extensibility. Moreover, fibrous cap strength in the proximal region (closer to the heart) was lower than that of the distal. These results are helpful in understanding the material behavior of atherosclerotic plaques.

## 1 Introduction

Despite significant advances in the diagnosis and management of stroke, it remains the third leading cause of death globally (WHO, 2008). Carotid atherosclerotic disease is responsible for 25–30% of all cerebrovascular ischemic events in western nations (Levy et al., 2008), with carotid luminal stenosis being the only validated diagnostic criterion for patient risk stratification. However, patients with mild to moderate carotid stenoses still account for the majority of clinical events (Barnett et al., 1998, Rothwell et al., 2003). Novel non-invasive screening methods are therefore urgently required to improve risk stratification of carotid plaques, in an attempt to avoid acute ischemic events. High-resolution, multi-sequence magnetic resonance (MR) imaging has shown great potential in identifying high-risk plaque morphological and compositional features, such as a large lipid-rich necrotic core, intraplaque hemorrhage (IPH) and fibrous cap defects with high accuracy and reproducibility (Chen et al., 2014, den Hartog et al., 2013, Touze et al., 2007). These MR-depicted plaque features have demonstrated their ability to differentiate patient clinical presentation (Grimm et al., 2013, Sadat et al., 2009) and for predicting subsequent ischemic cerebrovascular events (Altaf et al., 2008, Eliasziw et al., 1994, Singh et al., 2009, Takaya et al., 2006). However, IPH and FC rupture are both common in symptomatic lesions (prevalence of 60–70%) (Gao et al., 2007, Milei et al., 2003, Teng et al., 2014a), yet clinical recurrence rates are only 10–15% within the first year (U-King-Im et al., 2009). It is therefore clear that plaque morphological and compositional features alone, or in combination, cannot serve as a robust marker for prospective cerebrovascular risk, and additional analyses or biomarkers are required.

Studies have suggested that underlying pathological processes, such as inflammation and hypoxia, have an influence on plaque destabilization (Tarkin et al., 2014). Additionally, biomechanical factors are likely to play a role, as carotid atherosclerotic plaques are continually subject to mechanical loading due to blood pressure and flow. Plaque structural failure could occur when such loading exceeds its material strength. Therefore, biomechanical analyses may provide complementary information to luminal stenosis and plaque structure in determining vulnerability. Calculating mechanical stress within FC has been shown to differentiate patient clinical presentation both in the carotid (Sadat et al., 2010a, Sadat et al., 2011, Zhu et al., 2010) and coronary arteries (Teng et al., 2014a) and could provide incremental information to predict subsequent ischemic cerebrovascular events (Sadat et al., 2010b, Teng et al., 2013). These findings suggest that plaque morphological features and mechanical conditions should be considered in an integrative way, if plaque vulnerability assessment is to be improved.

To ensure that any mechanics-based vulnerability assessment is accurate, apart from the predicted mechanical loading within the plaque structure, the ultimate material strength and extreme extensibility of different atherosclerotic plaque components, including FC, media, lipid and IPH or thrombus (IPH/T) are needed. Available experimental data on this aspect is limited (Holzapfel et al., 2004, Mulvihill et al., 2013, Teng et al., 2009, Walsh et al., 2014) and hence we sought to quantify the ultimate material strength and extensibility of FC, media, lipid core and IPH/T in human carotid plaques from uni-extension tests in the circumferential direction.

## 2 Materials and methods

The local ethics committee approved the study protocol and all patients gave written informed consent. The patient demographics are shown in Table 1. Details of tissue preparation and testing and the equipment used have been described previously (Teng et al., 2014b). In brief, endarterectomy carotid plaque samples from 21 symptomatic patients were collected during surgery and banked in liquid nitrogen for <4 months prior to testing. Cryoprotectant solution added to a final concentration of 10% dimethylsulfoxide was utilized to prevent tissue damage from ice crystals formation and thawing (FAO, 2012). Prior to testing, samples were thawed in a 37 °C tissue bath and cut into 1–2 mm thickness rings perpendicular to the blood flow direction from proximal (closer to the heart) to distal, using a scalpel. Approximately 10 rings were obtained from each plaque. Each ring was further dissected to separate different atherosclerotic tissue components under a stereo microscope using fine ophthalmic clamps and scissors. The tissue strips were prepared carefully to minimize the variation of width and thickness along the length.

**Fig. 1:**
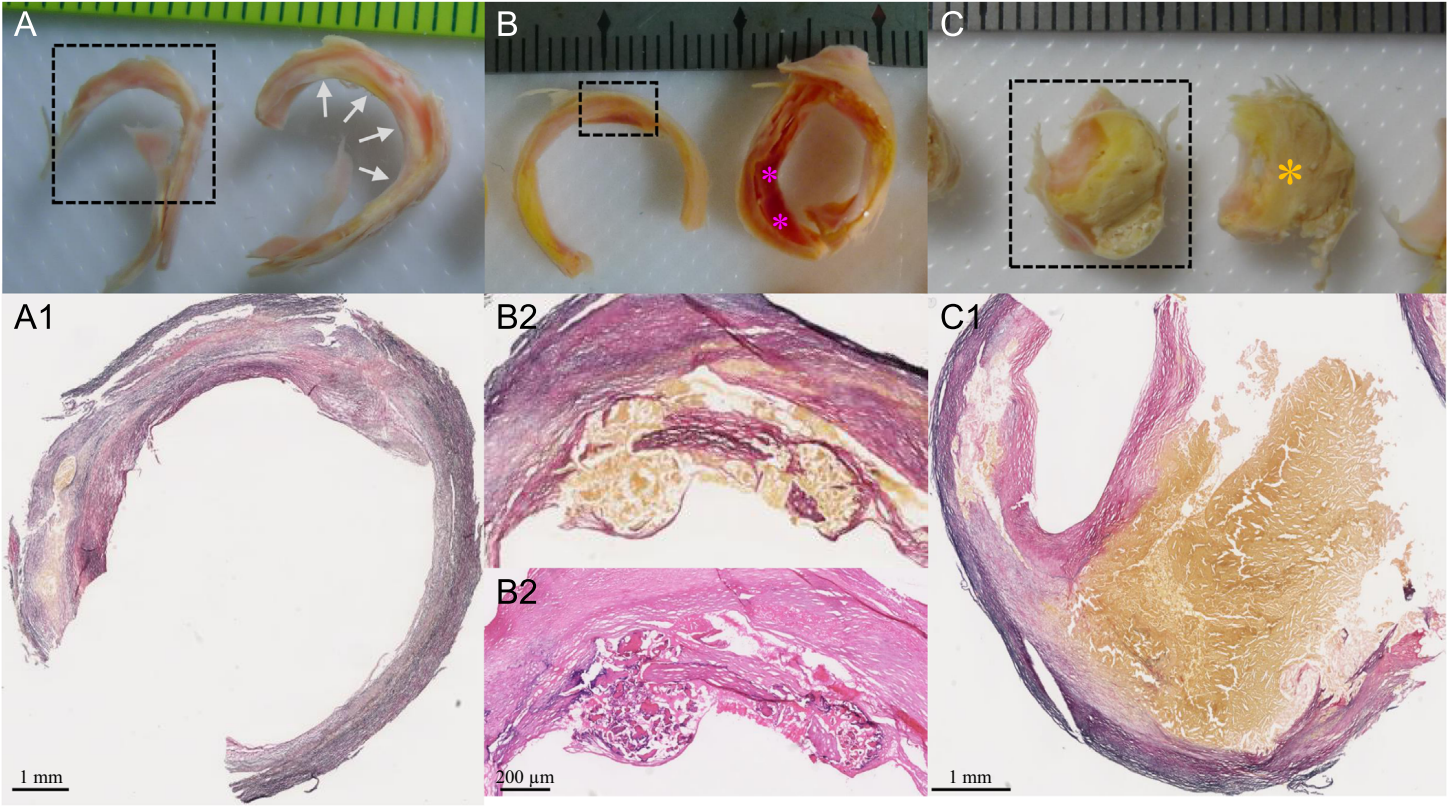
Tissue type characterisation and the corresponding histological examination (A and A1: the fibrous tissue in the ring enclosed by the dash box was confirmed by Verhoeff׳s Van Gieson (EVG) stain. This confirmed the inner layer (marked by gray arrows) of the ring next to it was fibrous cap; B, B1 and B2: intraplaque hemorrhage was located in the area enclosed by the dash box as confirmed by EVG and hematoxylin and eosin (H&E) stains. This confirmed the existence of intraplaque hemorrhage (marked by pink asterisks) in the ring next to it; C and C1: the big yellow block in the ring enclosed by the dash box was lipid crystals as shown in the EVG stains. This confirmed that the block marked by the brown asterisk was lipid crystals; rings pairs in A, B and C were adjacent to each other and the one enclosed by dashed boxes were for histological examination and the other for material testing). (For interpretation of the references to color in this figure legend, the reader is referred to the web version of this article.)

The identification and separation of each tissue component in atherosclerotic plaques is known to be challenging (Teng et al., 2014b). Prior to material testing, sample rings (rings enclosed by dash boxes in Fig. 1) adjacent to the ones used for material testing were chosen for training, assessing the accuracy of operators to identify and separate plaque component type through visual and histological means. As shown in Fig. 1A, the histological examination (Fig. 1A1) confirmed the fibrous nature of the inner layer marked by gray arrows in the ring next to it. Histological preparation of samples was performed following a standard protocol, with tissue strips being formalin-fixed and paraffin-embedded. Intraplaque hemorrhage appeared red/reddish within the wall as shown in Fig. 1B and confirmed by the histology as shown in Fig. 1B1 and B2. Thrombus typically appeared as a section of red/reddish substance obstructing the lumen. Lipid core was yellowish in color (Fig. 1C).

**Fig. 2:**
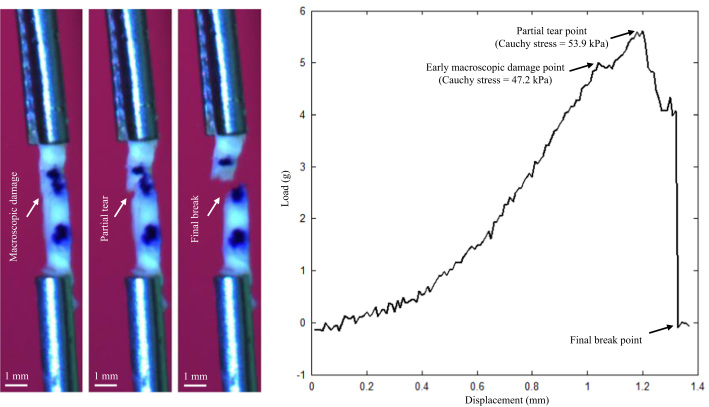
A lipid core strip under stretching and the corresponding load–displacement curve (this strip was eventually broke near the clamp).

**Fig. 3:**
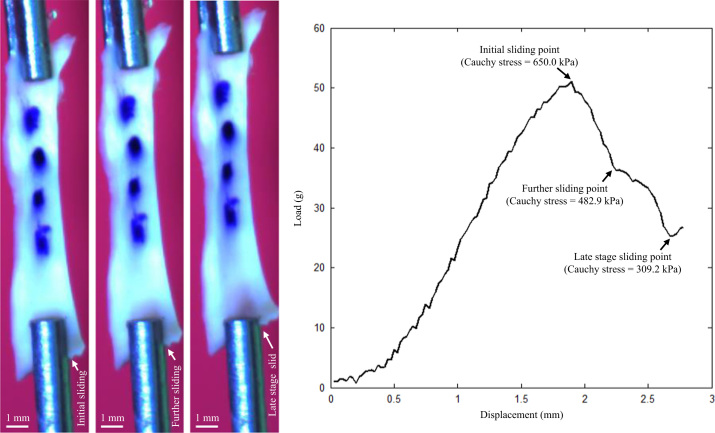
A media strip from a plaque under stretching and the corresponding load–displacement curve (this strip eventually slid away from the clamp).

An in-house designed tester, comprised of a stepper motor, load cell, camera and controlling system developed in LabView 2011 (National Instruments, USA) were used to perform uni-extension tests. The tissue strip was mounted on the tester by clamping at both ends with small pieces of sand paper attached. After five preconditioning cycles (about 2.5% stretching at a speed of 0.05 mm/s), the tissues strip was pulled at a speed of 0.01 mm/s (displacement control) with a 0.005 N pre-loading in a 37 °C saline bath until break (Fig. 2) or slide (Fig. 3) occurred. Waterproof black ink markers were placed on the surface to trace local displacement (Fig. 2, Fig. 3). The center of each marker was identified and the local stretch ratio was calculated from the distance between the centers. The Cauchy stress was computed from the measured force signal with the consideration of the strip thickness and width at rest and the stretch ratio with the material being assumed to be incompressible with the following definition,

$$\sigma =\lambda \frac{F}{A_{0}}$$

in which *σ* and *λ* stand for the Cauchy stress and stretch ratio, respectively; *F* is the stretching force; and *A*_0_ is the cross sectional area at rest, which can be approximated by the product of thickness and width at rest. The Cauchy stress and stretch ratio at the location with the peak loading adjacent to the sudden or steep drop of load–displacement curve were used to characterize the ultimate material strength and extensibility of each tissue strip. Tissue strips were deemed suitable for quantification of ultimate material strength if they tore either in the central region or at a location about 2 mm away from the clamp (Fig. 2).

As multiple measurements were obtained from each plaque, a linear mixed-effect model was used to assess the difference between parameters for different tissue types. All statistical analyses were performed in R 2.10.1 (The *R* Foundation for Statistical Computing), with statistical significant assumed when p value was <0.05. All results are presented as median [inter quartile range; *Q*1–*Q*3] for non-normally distributed data.

## 3 Results

In total, data from 32 FC strips from 12 samples, 35 media strips from 15 samples, 26 lipid core strips from 9 samples and 12 IPH/T from 7 samples qualified for analysis of ultimate strength and extreme extensibility. Data from tissue strips which slid or broke at the location close to the clamp were excluded (*n*=85). It is essential to minimize the variation of width and thickness along the tissue strip. The thickness, width and length of tissue strips included for analysis are: FC – 1.02±0.21, 1.71±0.48, 12.78±3.02; media – 0.97±0.34, 1.65±0.45, 15.44±4.72; lipid core – 1.17±0.33, 1.68±0.61, 8.92±2.17; and IPH/T – 1.24±0.40, 1.69±0.41, 9.07±3.51 (unit: mm).

**Fig. 4:**
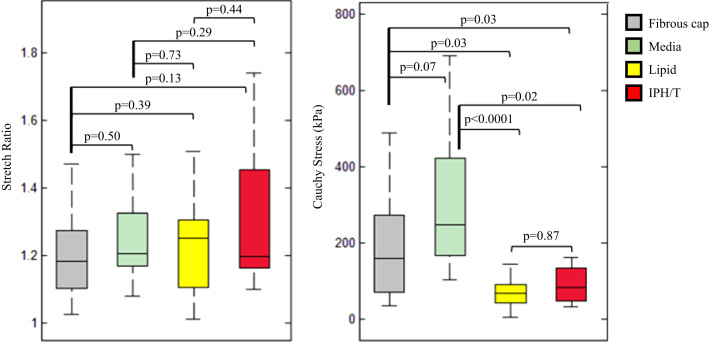
Comparisons of ultimate material strength and extensibility (A: extensibility of each tissue type; and B: ultimate material strength of each tissue type (one outlier in fibrous cap and one in media were not shown)).

The comparisons of ultimate material strength and extensibility of FC, media, lipid core, and IHP/T are presented in Fig. 4, with the exact values listed in Table 2. As shown in Fig. 4A, extreme extensibilities of each atherosclerotic tissues were comparable (*p*>0.05). But their ultimate strength differed (Fig. 4B). Both ultimate material strength of FC and media were comparable (*p*=0.07), as were that of lipid core and IPH/T (*p*=0.87). However, the ultimate material strength of both FC and media were significantly higher than either lipid core or IPH/T.

**Fig. 5:**
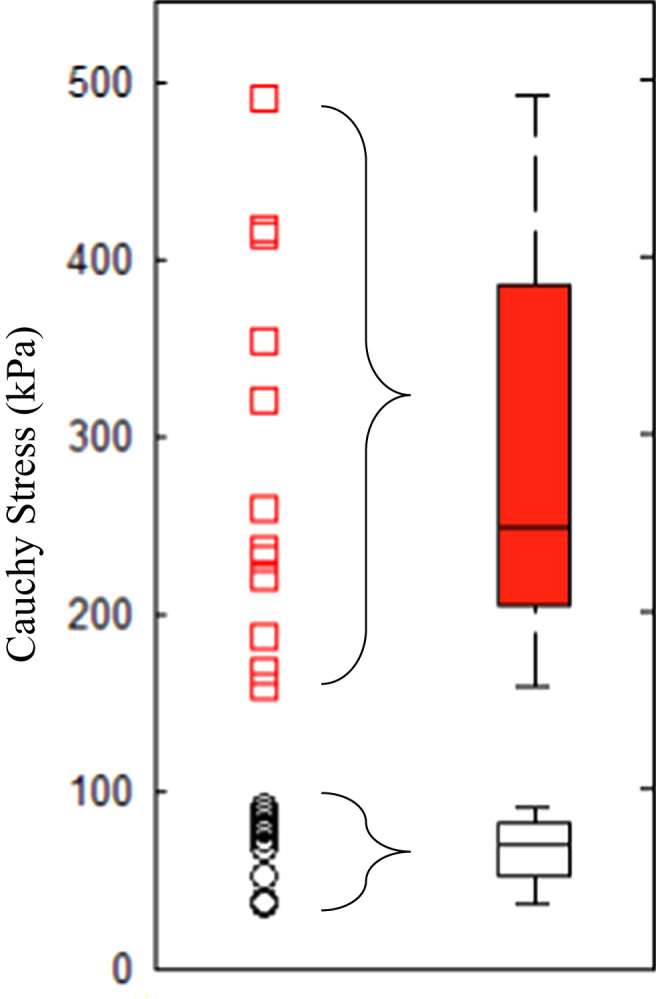
Data points showing ultimate strength of each fibrous cap strip, with a clear gap visible around 100 kPa (median [*Q*1–*Q*3] plot of both low (black circles) and high (red squares) strength groups is presented on the right). (For interpretation of the references to color in this figure legend, the reader is referred to the web version of this article.)

When each ultimate material strength value of FC was plotted vertically (Fig. 5), there appeared to be a visual separation into two groups, with ultimate material strength of 259.3 [220.4, 414.0] kPa for the stronger group and 69.7 [52.2, 80.2] kPa for the weaker group (data are presented as median [*Q*1–*Q*3]). 12/15 strips (80.0%) from the lower strength group were from the proximal plaque region (closer to the heart and the ring with the minimum ratio of luminal area to wall area being used as a reference), whereas 6/17 strips (35.3%) from the higher strength group were from this region (*p*=0.03). No such clear separation was found in media, lipid core and IPH/T.

**Fig. 6:**
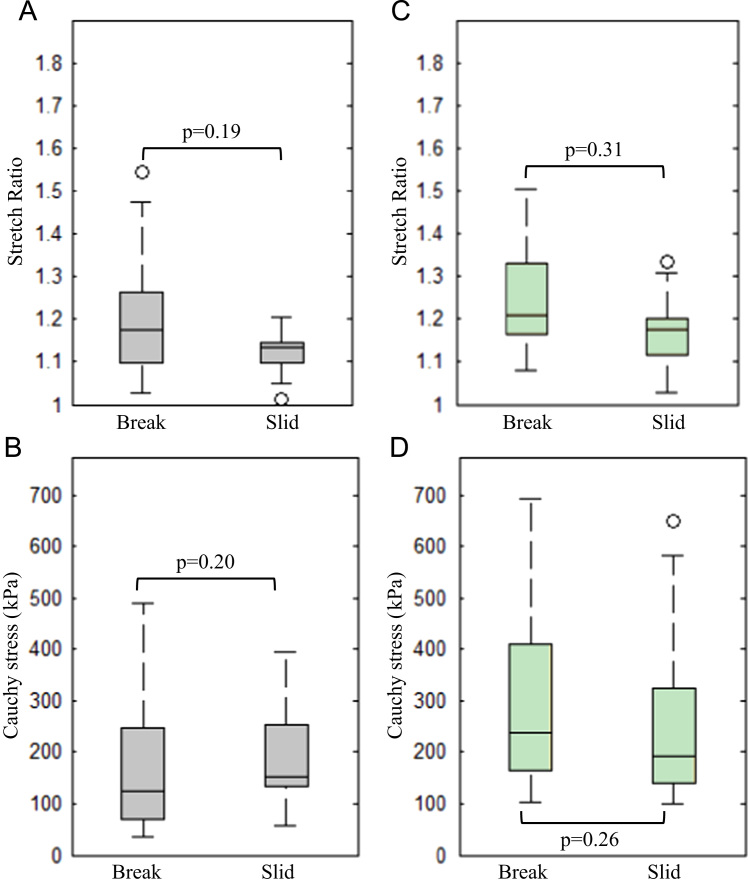
Box-and-whisker plots showing the comparison of ultimate strength and extensibility with the peak stress and stretch of strips slid away from the clamp (A and B: fibrous cap; C and D: media; the first row: extensibility; the second row: ultimate material strength; the box covered the 1st and 3rd quartiles and whiskers covered 99.3% data points; points beyond the whiskers were displayed using open circles, which however were not treated as outliers when the statistical analysis was performed).

The peak stress and stretch recorded from the strips which slid away indicated that those strips had an ultimate strength and extensibility above the recorded level. As shown in Fig. 6, no significant differences were found in FC and media in either extreme extensibility or ultimate strength (*p*>0.05). As no tissue strips of lipid core or IPH/T slid, such analysis was not performed with these.

## 4 Discussion

Our results show that FC and media have a similar ultimate material strength, as do lipid core and IPH/T. The ultimate material strength of FC and media are higher than either lipid core or IPH/T. Moreover, all atherosclerotic components have similar extreme extensibilities. Finally we observed that FC at the proximal region of the plaque is weaker than FC located distally.

It is important to understand the underlying mechanism of the clear separation of ultimate strength of FC as shown in Fig. 5. In the group with low strength, 80.0% strips were from the proximal region (closer to the heart), whereas only 35.3% strips were from the proximal region in the group with high strength. This observation may be due to the inherent difference in the pathological feature between the proximal and distal plaque region. It has been observed that the proximal region contains more macrophages and the distal contains more smooth muscle cells (Dirksen et al., 1998). The increased density of macrophages has been shown to reduce the material strength in FC in aortic atherosclerotic lesions, through release of matrix metalloproteinases (Lendon et al., 1991). A weaker FC in the proximal plaque region also is supported by the observation that the majority of angiography-defined carotid plaque ruptures/ulcerations (Lovett and Rothwell, 2003) are located in this region. In this study the most stenotic site defined as the minimum ratio of luminal area to wall area was used as a reference location, enabling the plaque to be divided into proximal and distal portions. The most stenotic site was determined by a visual estimation of luminal area and wall area. The lack of a fully quantitative approach may have introduced small errors in determining the exact lumen and wall area. In addition, the wall area mentioned here could not represent the total outer wall area of the lesion, as only parts of thickened intima or media were removed during the carotid endarterectomy.

To authors׳ best knowledge, only four studies (Lawlor et al., 2011, Maher et al., 2009, Mulvihill et al., 2013, Teng et al., 2009) have previously reported the ultimate strength and extreme extensibility of carotid atherosclerotic plaques. In three of these studies whole plaque specimens were classified according to their appearance on imaging before being subject to testing, while the other study separated plaque into two layers. The recorded failure features were therefore grouped and analyzed accordingly. In general, the stress level of failure varied within and between samples widely ranging from tens kPa to MPa and extreme extensibility varied from about 1.2 to 1.8. Results from these studies may be challenging to interpret though, as tissue components were not separated and tested. For example, in the mixture of lipid and fibrous tissues, the presence of soft lipid likely acts to destabilize the structure, reducing its ultimate material strength. The strength of our data is that we attempted to individually characterize the material properties of atherosclerotic tissue. Thus, researchers are able to use these data as the basis for computational simulations to predict rupture risk. However, the fact that material characteristics are location- and lesion-dependent should not be overlooked. Despite the heterogeneity between our methodology and previous studies, data on FC and media were remarkably similar between studies. There are studies reported the material strength and extreme extensibility of atherosclerotic lesion from other circulations, such as coronary, aorta and iliac artery, that have been summarized in a previous comprehensive review (Walsh et al., 2014). In this study, although some lipid core (Fig. 8A) and IPH/T were very fragile and unstable, some were strong and flexible as those shown in Fig. 2, Fig. 3, Fig. 8 in the reference (Teng et al., 2014b), permitting uni-axial testing (Fig. 8 B and C).

**Fig. 7:**
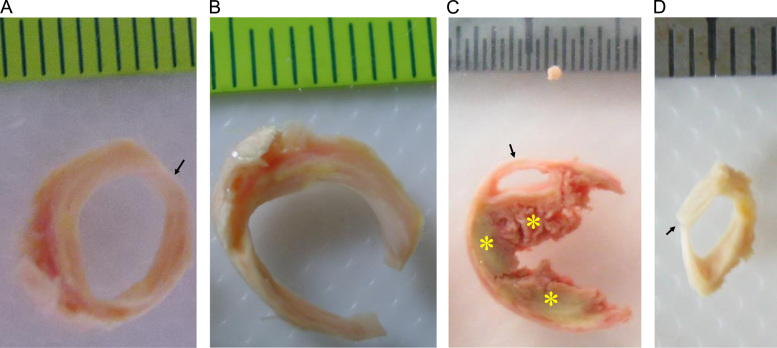
Residual stress in the atherosclerotic rings (A: an intact ring; B: the ring opened up to be a sector when a radial cut was make at the location marked with an arrow in A; C: a partial damaged ring with an intact lumen covered by a layer of fibrous cap (lipids were marked with yellow asterisks); D: The fibrous cap was isolated and the opening angle was not notable (arrow) suggesting very small or no residual stress existing in the cap (a radial cut was made at the location marked with an arrow in C)). (For interpretation of the references to color in this figure legend, the reader is referred to the web version of this article.)

In vivo imaging-based mechanical analysis in predicting stress/stretch has demonstrated its incremental value to predict subsequent ischemic cerebrovascular events (Sadat et al., 2010b, Teng et al., 2013). However, we should be cautious in combining the information of ultimate strength and extensibility obtained from direct material testing with computational modelling to assess FC rupture risk. Firstly, the effect of residual stresses (represented by an opening angle when a radial cut is made) in atherosclerotic plaques is unknown (Fig. 7). Although residual stress is rarely considered in the majority of in vivo imaging-based computation modelling studies, neglecting this force may lead to an overestimation of FC stress and stretch concentrations (Ohayon et al., 2007). Secondly, the ultimate strength has a close association with local inflammation, which may be quantifiable through ultrasmall superparamagnetic iron oxide-enhanced (USPIO) MR imaging (Tang et al., 2009) or positron emission tomography with 2-deoxy-2-[fluorine-18]fluoro-D-glucose integrated with computed tomography (^18^F-FDG-PET/CT) imaging (Tarkin et al., 2014). Lower strength thresholds should be adopted for mechanical-based vulnerability assessment if heavy inflammation is present within plaques. Thirdly, the ultimate material strength of FC obtained in study was from symptomatic patients. Some of these tissue strips, in particular those within the lower strength group shown in Fig. 5, might be from a location anatomically close to a previous rupture site. Therefore the median value of 70 kPa from this group should not be regarded as a universal threshold, while the median value of 260 kPa from the stronger group may be more appropriate in serving as a reference value for plaque regions with lower biological activity.

Our study also revealed evidence of tissue micro-damage (not visible) during the extension process that occurred much earlier than the final breakage, which is evidenced as steps shown along the load–displacement curves in Fig. 2, Fig. 3. Micro-damage occurred at a very low loading level as seen from the curves. These may be caused by high stress concentration due to uneven stretching or clamping, although inherent tissue imperfections, e.g., the presence of neovessels or voids due to cellular death, could also account for such early damage. This implies that under physiological conditions, tissues with abnormal configuration, such as large luminal curvatures induced by FC erosion and rupture, or structure, such as micro-calcium inclusions, could incur damage despite a relatively low stress/stretch level, resulting in repeated cycles of damage/healing leading to plaque progression.

**Fig. 8:**
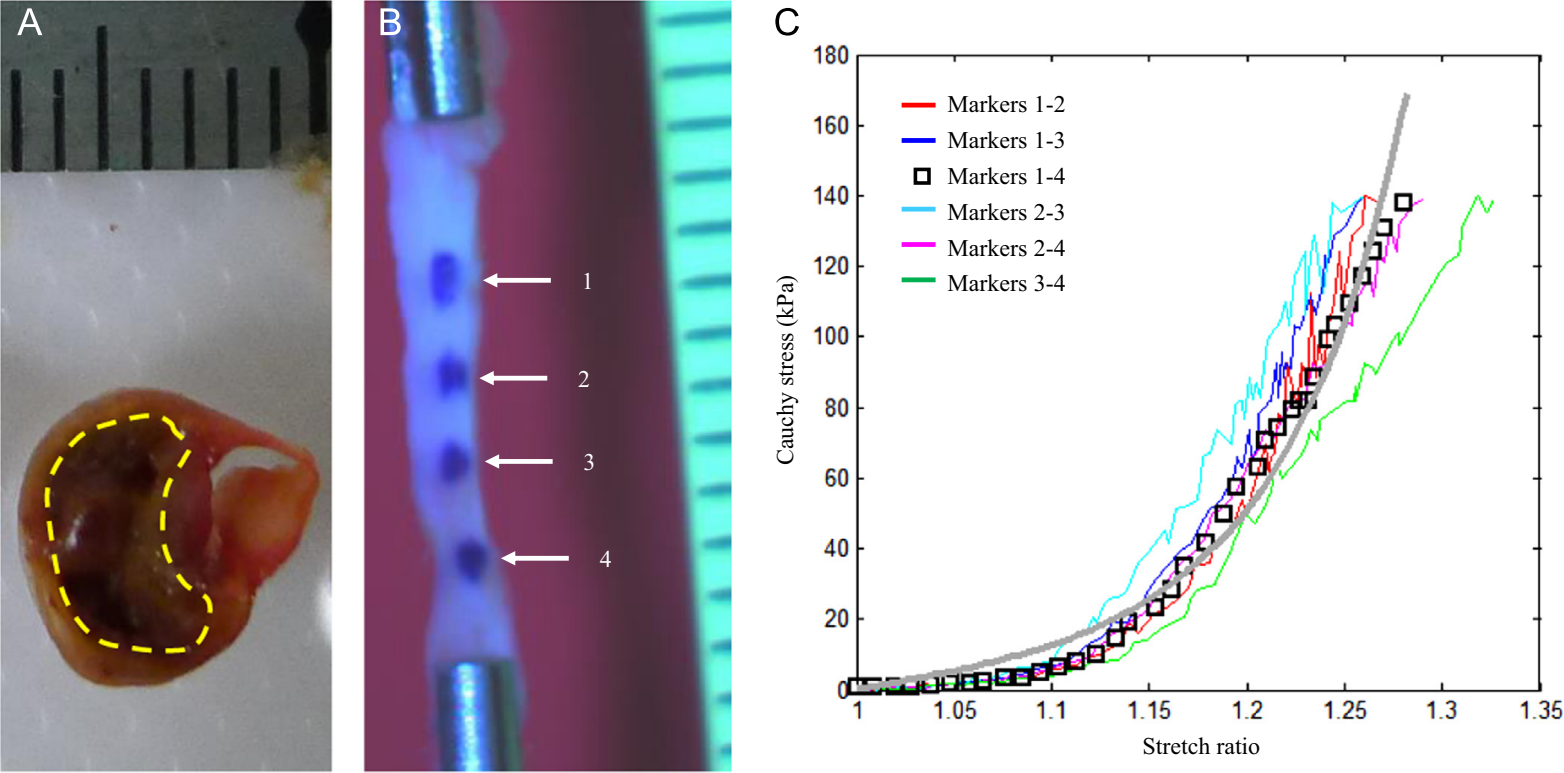
A fragile lipid core and a strip of intraplaque hemorrhage (A: the fragile lipid core enclosed by the yellow dashed line could dissolve away in the saline solution that made it inappropriate for extension testing; B: four markers were placed on the surface to trace the deformation of the strip of intraplaque hemorrhage; C: stretch–stress curves obtained by computing distance between each pair of markers showing in B; the length, width and thickness of this strip were (mm): 11.01; 1.67 and 1.17, respectively; a grid in the scale in A and B is 1 mm). (For interpretation of the references to color in this figure legend, the reader is referred to the web version of this article.)

In this study, the extreme extensibility was measured by using the stretch ratio, which is dependent on the marker location and the ratio of length to width/thickness. Ideally, the ratio of length to width/thickness should be over 10 and the markers needs to be located in the central region. However, due to small sample dimensions, it was difficult to meet these ideal criteria. In this study, the length to width ratios for FC and media were about 7 and those for lipid core and IPH/T were smaller, at around 5. The length to thickness ratios for FC and media were >10 and those for lipid core and IPH/T were ~7. Although these ratios are not ‘perfect’, they should be acceptable for material testing. As shown in Fig. 8B and C, the difference of stress–stretch curves obtained by tracing the distance between different pairs of the 4 markers placed along the strip was small. This implies that the deformation in the region about 1–2 mm away from the clamp should be reasonably uniform when the ratio of length to width or thickness is about 5–7.

**Fig. 9:**
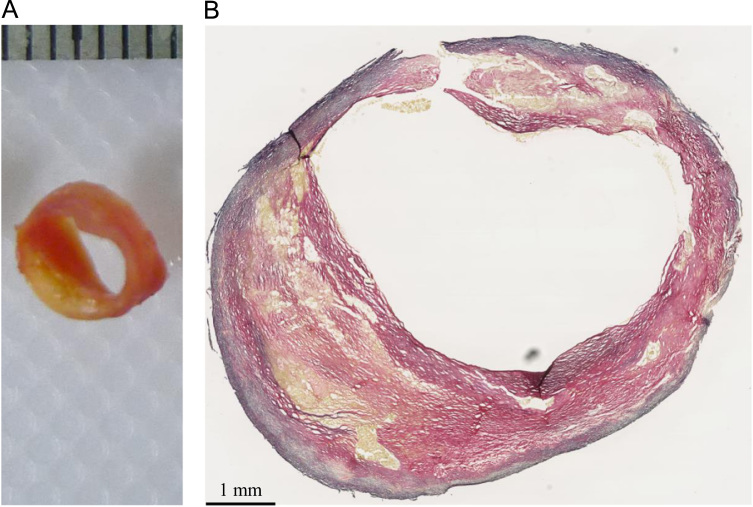
The yellowish block (A) in the atherosclerotic plaque might not be pure lipid, but the mixture of lipid and fibrous tissues (B). (For interpretation of the references to color in this figure legend, the reader is referred to the web version of this article.)

Despite our robust methodology, there are limitations to this study; (1) due to small sample dimensions, only the strength and extensibility in the circumferential direction were quantified; (2) there is likely to be a close relationship between strength/extensibility and tissue microstructure, e.g., presence of micro calcium (Vengrenyuk et al., 2006) and fiber orientation. However, following testing the tissue strips became contaminated and accordingly, such information was no longer available; (3) calcium was not tested in this study; (4) some tissue strips may contain more than one tissue type due to the highly heterogeneous nature of atherosclerotic plaques. As shown in Fig. 9, the yellowish block would be judged to be lipid core, however it was mixture of lipid and fibrous tissues when reviewed under light microscopy. Although the authors attempted to dissect tissue strips by careful inspection using a stereo microscope, potential tissue misclassification still remained; (5) the tissue type was discriminated according to the appearance of the slice used for testing and the corresponding histology of an adjacent slice. Misclassification could potentially occur if tissue type changed dramatically in a short longitudinal distance; moreover in this study, strips of lipid core were from big yellowish tissue blocks, most of which should be true lipid cores (Fig. 1C). However, as shown in Fig. 9 that this statement might not hold in some of them; and (6) cryoprotectant solution was used to prevent tissue damage from ice crystals formation. This storage approach may slightly alter mechanical behavior of atherosclerotic tissue due to solution permeation into the tissue and lipid extraction.

## 5 Conclusions

The ultimate material strength of atherosclerotic components differs with FC and media being comparable, as were lipid and IPH/T. All tissue subtypes exhibited similar extensibility.

## Disclosure

The authors do not have any conflict of interest to be declared.

## Figures and Tables

**Table 1:** Patient demographics (*n*=21).

Male, *n* (%)	18 (85.7)
Age, (Mean±SD)	68.2±7.4
Hypertension, *n* (%)	19 (90.5)
Coronary artery disease, *n* (%)	6 (28.6)
Diabetes mellitus, *n* (%)	4 (19.0)
Previous use of statin, *n* (%)	12 (57.1)
NASCET defined stenosis, (%)	72.3±17.0%

**Table 2:** The ultimate material strength and extreme extensibility of different tissue types in the circumferential direction (median [*Q*1–*Q*3]).

	Ultimate strength (kPa)	Extreme extensibility
Fibrous cap (FC)	158.3 [72.1, 259.3]	1.18 [1.10, 1.27]
Media	247.6 [169.0, 419.9]	1.21 [1.17, 1.32]
Lipid	68.8 [48.5, 86.6]	1.25 [1.11, 1.30]
Intraplaque hemorrhage/thrombus (IPH/T)	83.0 [52.1, 124.9]	1.20 [1.17, 1.44]
